# Reperfusion failure after successful thrombectomy of large vessel occlusion stroke: clinical and imaging evidence

**DOI:** 10.3389/fneur.2025.1639880

**Published:** 2025-08-22

**Authors:** M. Barbagallo, M. Zahn, J. Zimmermann, R. Klövekorn, J. Held, B. Nemeth, B. Reolon, J. Bellomo, A. Schwarz, J. M. Veerbeek, C. H. B. Van Niftrik, M. Sebök, M. Piccirelli, L. Michels, A. R. Luft, Z. Kulcsar, L. Regli, G. Esposito, J. Fierstra, P. Thurner, T. Schubert, S. Wegener

**Affiliations:** ^1^Department of Neurology, University Hospital Zurich, University of Zurich, Zurich, Switzerland; ^2^Clinical Neuroscience Center, University of Zurich, Zurich, Switzerland; ^3^Lake Lucerne Institute, Vitznau, Switzerland; ^4^Universitätsklinik für Radiologie und Nuklearmedizin Universität Wien, Zurich, Switzerland; ^5^Faculty of Information Technology and Bionics, Pazmany Peter Catholic University, Budapest, Hungary; ^6^Department of Neuroradiology, University Hospital Zurich, Zurich, Switzerland; ^7^Department of Neurosurgery, University Hospital Zurich, Zurich, Switzerland; ^8^Department of Neurology, David Geffen School of Medicine at University of California, Los Angeles, CA, United States; ^9^Clinic for Neurology and Neurorehabilitation, Lucerne Cantonal Hospital, University Teaching and Research Hospital of the University of Lucerne, Lucerne, Switzerland; ^10^Cereneo Center for Neurology and Rehabilitation, Vitznau, Switzerland

**Keywords:** reperfusion failure, futile recanalization, stroke, perfusion study, stroke thrombectomy

## Abstract

**Introduction:**

Reperfusion failure (RF) describes a condition in which patients suffering a large vessel occlusion (LVO) stroke present insufficient tissue reperfusion and recovery despite optimal mechanical thrombectomy (MT) results. Approximately 50% of patients suffering from LVO are affected. Our current understanding of the underlying pathomechanisms is limited and mostly based on rodent models. The goal of this study was to further characterize RF by applying advanced multimodal hemodynamic imaging in stroke patients.

**Methods:**

Patients from the IMPreST study with LVO stroke and successful recanalization [corresponding to thrombolysis in cerebral ischemia grade (TICI) 2b-3] were included. Follow-ups with blood oxygenation-level dependent cerebrovascular reactivity (BOLD-CVR) and non-invasive optimal vessel analysis (NOVA) imaging were performed (<72 h, 7 days and 90 days). Demographic and clinical data (NIHSS and mRS) were collected.

**Results:**

Of the 49 patients included in IMPreST, 18 patients met the inclusion criteria. Based on the perfusion weighted imaging (PWI) of the affected area compared to the contralateral side after MT, patients were stratified into three groups: hypoperfusion (*n* = 3), normalization (*n* = 8), and hyperperfusion (*n* = 7). The hyperperfusion group tended to show poorest clinical outcome (mRS 3 months: 2.5 [Q1–Q3 2.0–3.0] vs. normalization: 1 [Q1–Q3 0.75–3.0], *p* = 0.169) and had significantly lower BOLD-CVR values at visit one and two compared to hypoperfusion and normalization groups, indicating impaired cerebrovascular reactivity (visit1 hyperperfusion group −0.01 [Q1–Q3–0.02 – 0.07], normalization group 0.12 [0.09, 0.19], hypoperfusion group, 0.09 [0.09, 0.11] *p* = 0.049, visit2 hyperperfusion group 0.07 [Q1–Q3 0.03–0.10], normalization group 0.17 [0.16, 0.18], hypoperfusion group 0.10 [0.09, 0.11], *p* = 0.014).

**Discussion:**

We found three patterns of reperfusion after successful MT of LVO stroke: normalization, hypo- and hyperperfusion of the ischemic area on days at < 72 h after stroke. There was substantial inhomogeneity in perfusion and clinical outcomes between the three groups. Next to poorest clinical outcome, the hyperperfusion-group showed poorest cerebrovascular reserve, reflecting findings of RF in rodent models. Thus, we suggest that RF includes both hypo- as well as hyperperfusion. Early detection using advanced imaging would allow a better identification of patients at risk for poor clinical outcome.

**Clinical trial registration:**

http://clinicaltrials.gov, Identifier (NCT04035746).

## 1 Introduction

Stroke is a major global health burden, representing the third-leading cause of death and disability combined worldwide (1). Despite continuous improvements in the treatment of large vessel occlusion strokes (LVO) including mechanical thrombectomy (MT) (2), there is still a substantial number of patients with insufficient clinical recovery despite optimal recanalization therapy. This phenomenon is known as futile recanalization and likely due to “reperfusion failure” (RF), i.e., inadequate brain tissue reperfusion despite recanalization, and affects more than 50% of patients with LVO and restored circulation, fulfilling the criteria for thrombolysis in cerebral infarction (TICI) of 2b-3 after MT (3–5).

The mechanisms underlying RF are not clarified yet. Currently, studies indicate that dysfunctional vascular regulation might play a key role (6, 7). Based on observations in rodent models, different components have been described: first, at a macrovascular level, a malfunction in the vascular contractility with consecutive constriction and rigidity of the large vessels and/or capillaries (8). Second, at a microvascular level, intraluminal occlusion provoked by distal embolization of dissolved clots and development of micro-clots or stalls with neutrophils (7, 9, 10).

While the suggested mechanisms of RF are primarily based on observations from rodent stroke models, knowledge confirming the concept in stroke patients with LVO is limited. In a study with patients suffering from LVO, presenting within 4.5 h after onset, about 25% of patients with recanalization result TICI 2c-3 presented regions of persistent hypoperfusion on cerebral blood volume (CBV) maps within the infarct region on post-CT or MRI perfusion imaging (11). This was associated with a higher rate of hemorrhagic transformation, larger infarct growth and worse clinical outcome, along with worse national institutes of health stroke scale (NIHSS) at 24 h and modified Ranking score (mRS) at 90 days after onset. Similarly, a systematic review confirmed that about a third of patients with macrovascular reperfusion showed signs of microvascular impairment, resulting in a reduced rate of functional independence. However, it remains unclear whether the phenomenon is merely an epiphenomenon of the infarcted parenchyma or if it contributes to further infarction (12).

Considering the large number of affected patients, a better characterization of RF is needed in order to derive targeted approaches for normalizing reperfusion and improving outcome after stroke treatments.

In this study, we used advanced multimodal hemodynamic imaging to describe different patterns of RF in patients with LVO stroke after MT, allowing a comprehensive view on the macro- and microvascular perfusion in affected patients.

## 2 Materials and methods

This study was conducted as a sub-analysis of the interplay of microcirculation and plasticity after ischemic stroke (IMPreST) trial. IMPreST (clinicaltrials.gov, No. NCT04035746) is a prospective, longitudinal, and observational cohort study, with the goal to illuminate the interplay between the microcirculation and clinical outcomes after LVO stroke based on serial perfusion and hemodynamic imaging studies and to better understand the plasticity of brain tissue after stroke. Data was collected between October 2018 and March 2022 at the University hospital of Zurich, Switzerland. The study was approved by the ethics commission of the canton of Zurich, Switzerland (Kantonale Ethikkommission Zürich, KEK-ZH-NR. 2019–00750). The study adheres to the Declaration of Helsinki of 1964.

### 2.1 Patient selection and consent to participate

Within the IMPreST study, patients presenting an acute first-ever hemispheric LVO and undergoing triage for an endovascular acute stroke treatment were eligible. Inclusion criteria were: 72 h first clinical ischemic stroke at hospital admission, occlusion of a M1/M2-segment of the middle cerebral artery and/or intracranial internal carotid artery, perfusion deficits with cortical involvement, 18 years of age or above, living independent before stroke (corresponding to a mRS of ≤ 3), written informed consent of the patient or a documented authorization of an independent doctor, not involved in the study, or a *post-hoc* written informed consent of the patient or next of kin. Exclusion criteria were age under 18 years, contraindication to MRI, and presence of major neurological, psychiatric, or medical comorbidities (such as major cardiac, psychiatric and/or neurological diseases, early seizures, known or suspected non-compliance, drug and/or alcohol abuse, contra-indications for MRI). For this sub-analysis, only patients fulfilling the criteria of TICI of 2b-3 after MT with available perfusion studies at onset and <72 h after onset were considered.

### 2.2 Image acquisition

The imaging data included a pre-interventional CT or MRI scan within 24 h after clinical onset. The CT perfusion was performed respecting the standard-of-care protocols at the University Hospital Zurich. The modalities diffusion weighted imaging (DWI) and contrast-enhanced perfusion MRI were acquired post-MT. Follow-up scans were performed <72 h (visit one), 7 days (+/− 2 days; visit two) and 90 days (+/− 14 days; visit three) after onset using a 3-Tesla Skyra MRI scanner (Siemens Healthineers, Forchheim, Germany).

Within the MRI protocol, the T1 magnetization-prepared rapid gradient echo (MPRAGE), followed a repetition and echo time (TR/TE) OF 2200/5.14 ms, slice thickness of 1 mm, voxel size 1x1x1 mm3, flip angle 8°, field of view of 230 mm. The DWI included 2D EPI sequence, TR/TE of 2500/75 ms, flip angle by 90°, slice thickness of 4.5 mm, voxel size with 1.5 × 1.5 × 4.5 mm3, b-values of 0 and 1000s/mm2. For the MR PWI the following protocol was applied: TR/TE of 2040/36 ms, flip angle of 90°, slice thickness of 4 mm, voxel size 1.7 × 1.7 × 4.0 mm3, FOV of 220 mm. Other MRI sequences were acquired as part of the established imaging protocol. These were not included in the analysis for this study.

For the acquisition of the blood oxygenation-level dependent cerebrovascular reactivity (BOLD-CVR) MRI sequences, standardized CO_2_ impulse was applied by RespirAct™ (Thornhill Research Institute, Toronto, Canada), allowing targeted CO_2_ end-tidal pressure (P_et_CO_2_) while maintaining iso-oxic O_2_ level (13). The used CO_2_ protocol in our institute followed a 100 s phase of patient specific resting P_et_CO_2_, whereafter the P_et_CO_2_ was increased by 10 mmHg for the duration of 80s, after which it returned to the resting P_et_CO_2_ for 120 s.

In the case of contrast-enhanced perfusion imaging, Dotarem Gadoteric acid (Gadoterate meglumine) from Guerbet, Villepinte, France, was administered intravenously with a dosage of 0.2 mL/kg body weight at a flow of 5 mL/s. Thereafter, a saline flush between 25 and 30 mL was applied to ensure full circulation of the contrast agent.

### 2.3 Image processing

Perfusion weighted imaging (PWI) included a cerebral blood flow (CBF), cerebral blood volume (CBV) and a time-to-maximum (Tmax) analyses calculated applying RAPID^®^ software (14). Based on the perfusion parameter maps post MT, patients were categorized qualitatively by three authors (MB, MZ and SW) into three different reperfusion groups (normalized, hypo-, and hyperperfusion).

Apparent diffusion coefficient (ADC) maps were calculated applying Bayesian estimation with the RAPID® software.

For the BOLD-CVR imaging processing we followed the previously described Zurich analysis pipeline (15) using MATLAB2019 (The MathWorks, Inc., Natrick, United States) and SPM12 (Welcome Trust Center for Neuroimaging, Institute of Neurology, University College London). The BOLD-CVR were calculated voxel-per-voxel as percentage of BOLD signal change, divided by the absolute change in P_et_CO_2_ (% ΔBOLD/mmHg).

### 2.4 Clinical characteristics

Clinical characteristics such as age, sex, vascular risk factors, time-to-recanalization, location of the occlusion, application of intravenous thrombolysis (IVT), NIHSS on admission, 24 h and 3 months after onset and mRS at admission, 36 h and 3 months after onset were collected and analyzed.

### 2.5 Categorization of perfusion group

Based on the colored map of the Tmax, CBF and CBV parameters at visit one (<24 h after onset), the patients were categorized into one of the following groups: hyperperfusion, if Tmax was shorter and CBF and CBV higher compared to the contralateral hemisphere; hypoperfusion, if Tmax was longer and CBF and CBV lower compared to the contralateral hemisphere; normalized, if Tmax, CBF and CBV were comparable to the contralateral side. The comparison of the perfusion parameters was focused on the region within the infarct and the penumbra region. The selection was performed by three authors (MB, MZ, SW). Discrepancies in the selection process were resolved through discussion.

### 2.6 Statistical analysis

Descriptive statistics were used to compare the demographics, clinical patterns, therapeutic modalities and advanced perfusion study parameters between the perfusion groups. Ordinal, non-dichotomous variables are presented as median and quartiles one and three (Q1–Q3). Categorical dichotomous and non-dichotomous variables are presented as percentages. Assuming a nonnormal distribution, the Kruskal-Wallis-Test was applied to compare the variables. We defined the level of significance as the probability for a type I error of less than 5%, corresponding to a *p*-value of <0.05.

All statistical analysis were performed in R, version 4.3.0.

## 3 Results

**Figure 1:**
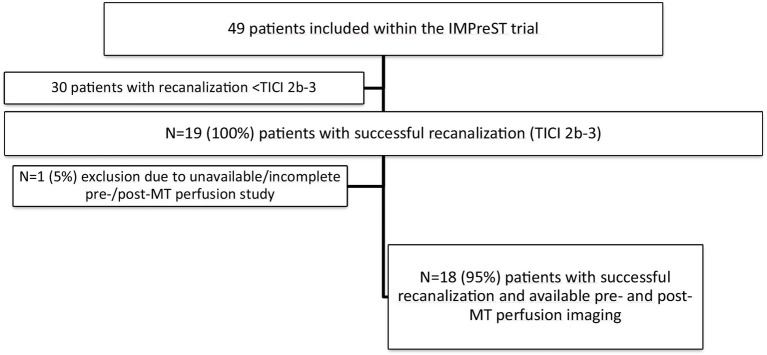
Overview of patient selection: Of the 49 patients enrolled in the IMPreST study, 19 achieved recanalization equivalent to TICI 2b 3. Additionally, 18 patients provided complete pre- and post-MT perfusion studies. MT, Mechanical Thrombectomy; TICI, Thrombolysis in Cerebral Infarction.

Of the 49 patients enrolled within the IMPreST study, we identified 19 patients fulfilling the criteria of TICI 2b-3 after MT. 18 patients had available pre- and postinterventional PWI and were included in the analysis (Figure 1).

**Figure 2:**
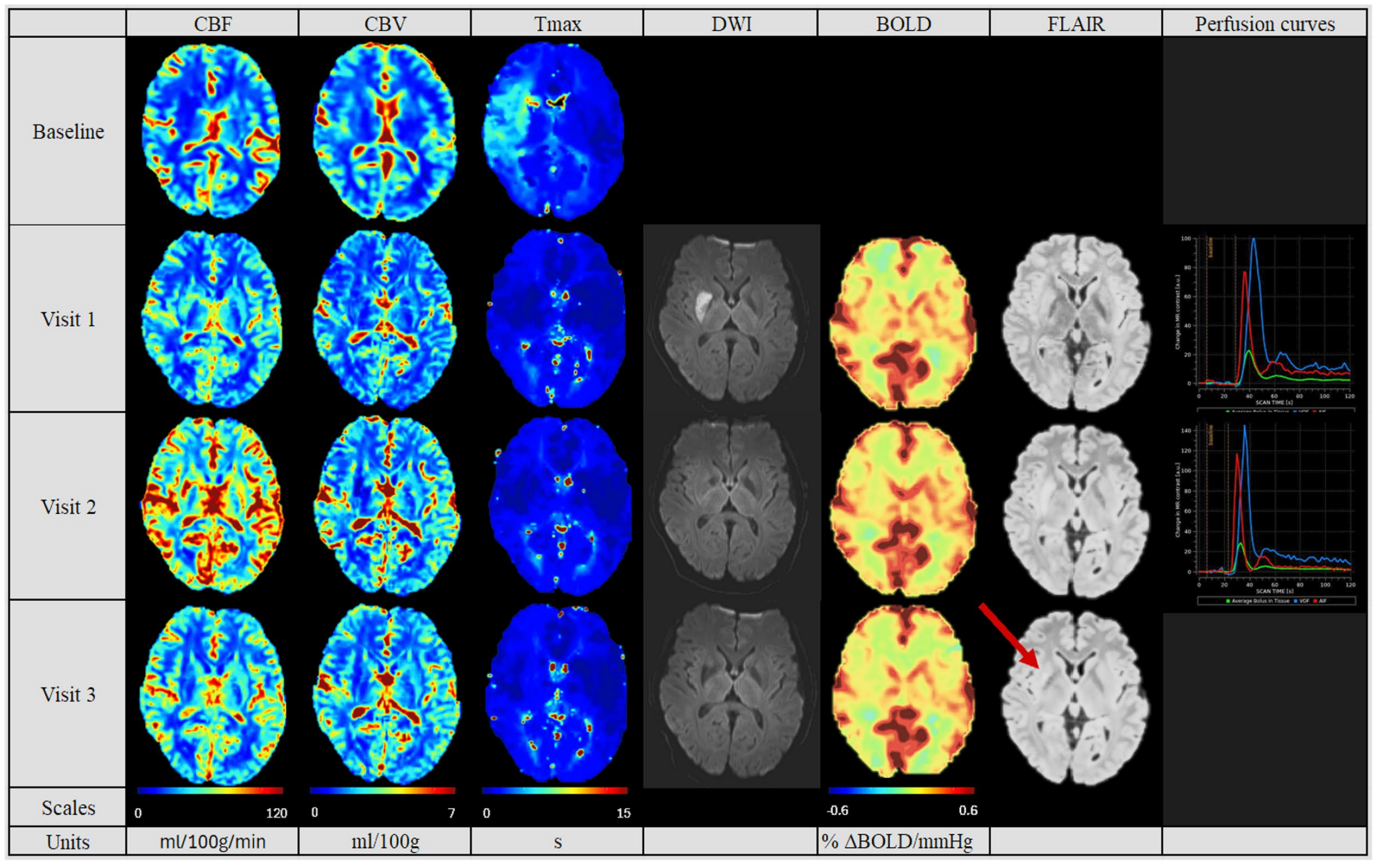
Perfusion pattern “normalized”. In this perfusion group, patients showed after MT similar CBF, CBV and Tmax values in both hemispheres. In addition, perfusion studies including BOLD-CVR, DWI and FLAIR modalities are shown. Finally, perfusion curves show the change in contrast agent over time, where the red line indicates the AIF, the blue line the VOF and the green line the average bolus in tissue (no V3 available). In this group, no perfusion dynamics could be observed. The FLAIR hyperintense signal showed the final lesion volume (indicated by the red arrow). Visit 1 was performed <72 hours, Visit 2 7 ± 2 days, and Visit 3 90 ± 14 days after onset. AIF: Arterial Input Fraction, BOLD-CVR: Blood Oxygenation-Level dependent Cerebrovascular Reactivity, CBF: Cerebral Blood Flow, CBV: Cerebral Blood Volume, DWI: Diffusion Weighted Imaging, FLAIR: Fluid Attenuated Inversion Recovery, MT: Mechanical Thrombectomy, Tmax: Time-to-Maximum, VOF: Venous Output Function.

**Figure 3:**
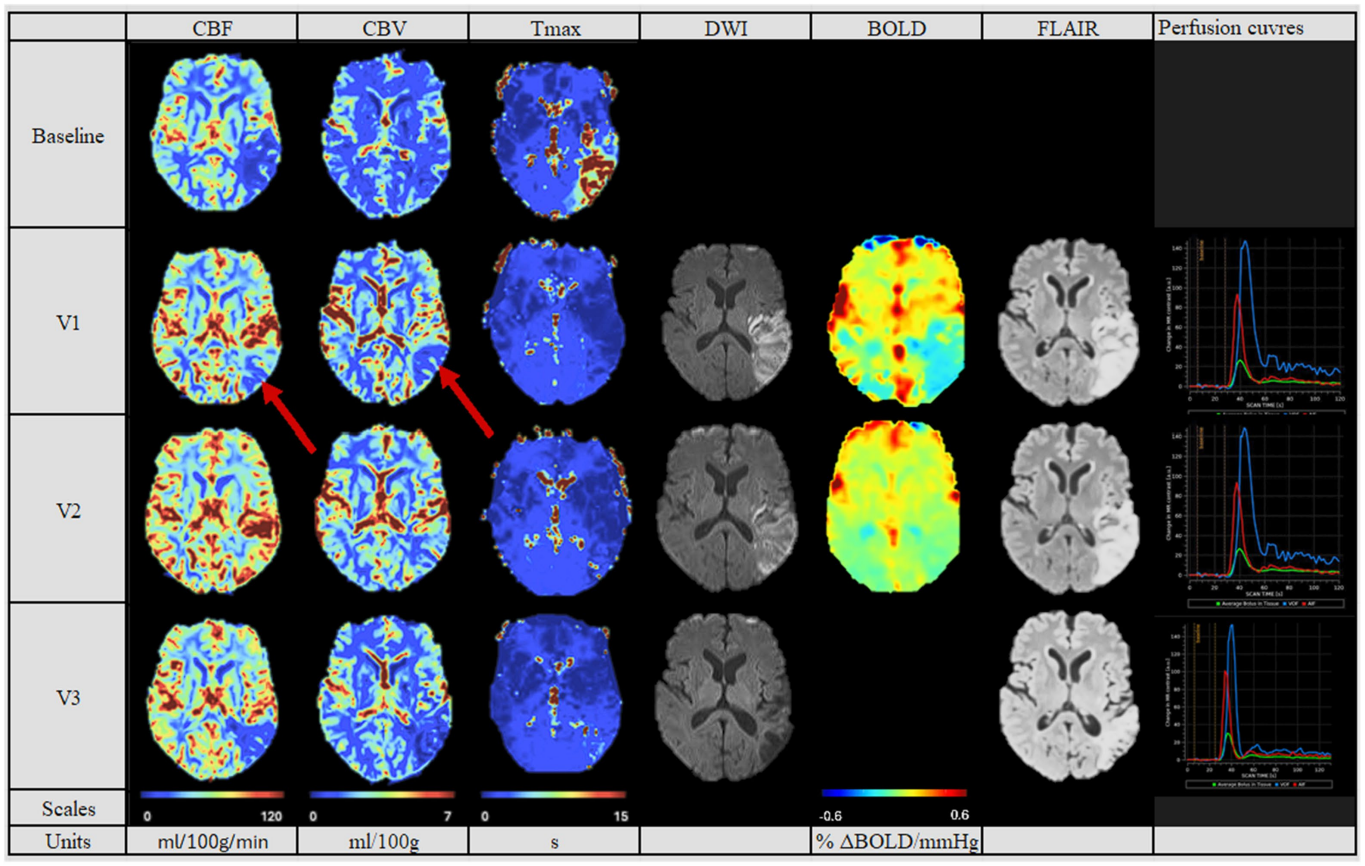
Perfusion pattern “hypoperfusion“: In this perfusion group, patients exhibited after MT a persistently diminished perfusion of the affected side based on CBF, CBV and Tmax values. The difference compared to the contralateral side is pronounced. In addition, perfusion studies including BOLD-CVR (no Visit 3 available), DWI and FLAIR modalities are shown. Finally, perfusion curves show the change in contrast agent over time, where the red line indicates the AIF, the blue line the VOF and the green line the average bolus in tissue. The BOLD-CVR showed persistently slightly diminished values. DWI and FLAIR modalities indicated the persistently compromised brain tissue, reflected in the second largest lesion size of the cohort. The hypoperfused area in Visit 1 on the CBV and CBF maps is indicated by red arrows. Visit 1 was performed <72 hours, Visit 2 7 ± 2 days, and Visit 3 90 ± 14 days after onset. AIF: Arterial Input Fraction, BOLD-CVR: Blood Oxygenation-Level dependent Cerebrovascular Reactivity, CBF: Cerebral Blood Flow, CBV: Cerebral Blood Volume, DWI: Diffusion Weighted Imaging, FLAIR: Fluid Attenuated Inversion Recovery, MT: Mechanical Thrombectomy, Tmax: Time-to-Maximum VOF: Venous Output Function.

**Figure 4:**
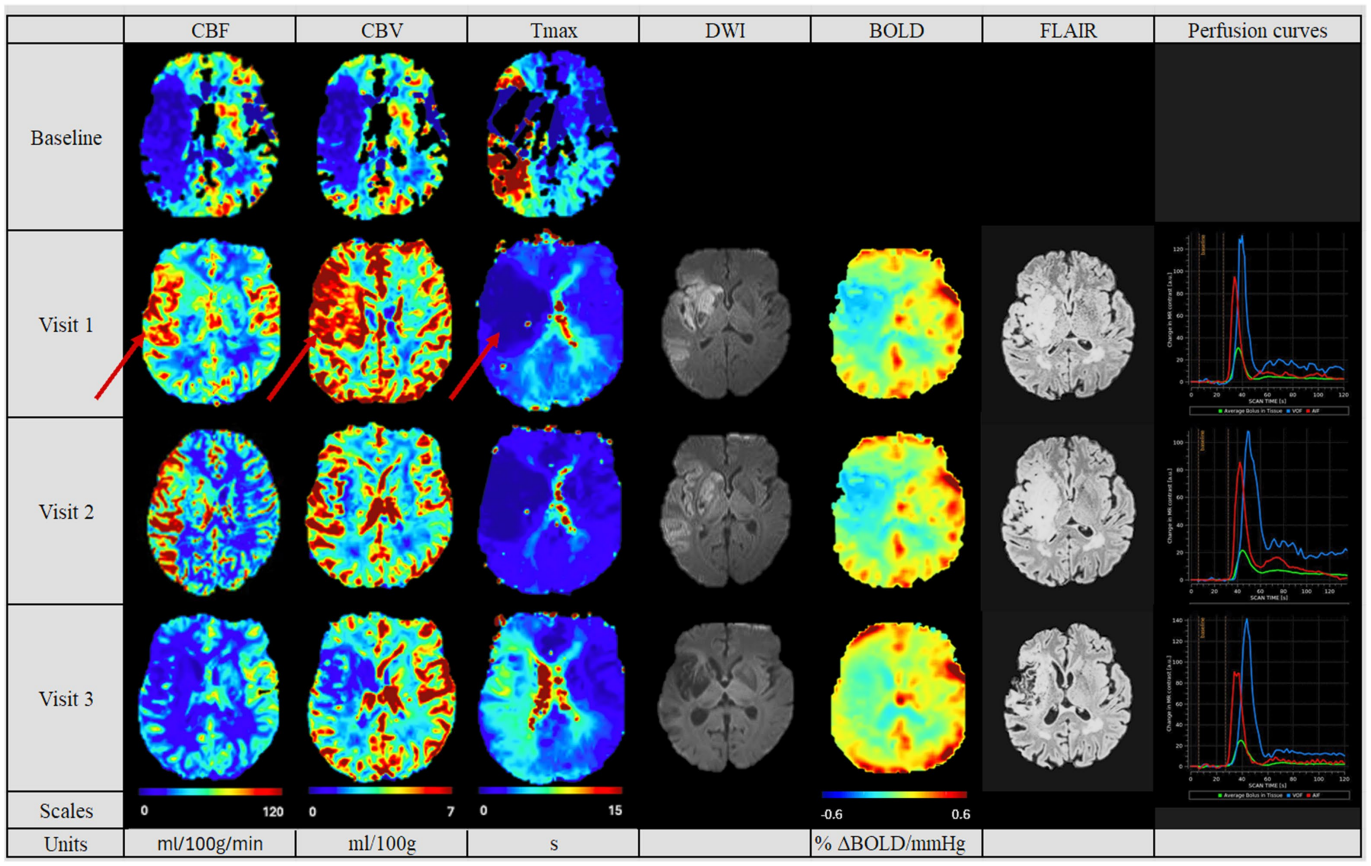
Perfusion pattern “hyperperfusion“: In this perfusion group, patients exhibited after MT an increased perfusion of the affected side at the first follow-up. The hyperperfused area in Visit 1 on the CBV, CBF and Tmax maps is indicated by red arrows. The CBF, CBV and Tmax values continuously decreased in the follow-ups. In addition, perfusion studies including BOLD-CVR, DWI and FLAIR modalities are shown. Finally, perfusion curves show the change in contrast agent over time, where the red line indicates the AIF, the blue line the VOF and the green line the average bolus in tissue. The BOLD-CVR showed persistently diminished values. DWI and FLAIR indicated the largest lesion size of the cohort. Visit 1 was performed <72 hours, Visit 2 7 ± 2 days, and Visit 3 90 ± 14 days after onset. AIF: Arterial Input Fraction, BOLD-CVR: Blood Oxygenation-Level dependent Cerebrovascular Reactivity, CBF: Cerebral Blood Flow, CBV: Cerebral Blood Volume, DWI: Diffusion Weighted Imaging, FLAIR: Fluid Attenuated Inversion Recovery, MT: Mechanical Thrombectomy, Tmax: Time-to-Maximum VOF: Venous Output Function.

Considering the post-MT PWI, the patients showed three different patterns of reperfusion after MT: Eight patients had a normalized perfusion, in which the CBF, CBV and Tmax values were similar to the non-affected contralateral hemisphere (see Figure 2). Three patients had diminished perfusion parameters compared to the contralateral hemisphere (see Figure 3) and were categorized into the “hypoperfusion” group. Finally, seven patients showed increased perfusion compared to the contralateral side, thus representing the “hyperperfusion” group (see Figure 4). In addition, the perfusion curves showed diminished values after Visit one in the case of the hyperperfusion group, while they stayed comparable or increased slightly in the other two groups (compare Figures 2–4).

The demographical and clinical characteristics, as well as the treatment modalities of all patients are summarized in Tables 1, 2. The group with the normalized perfusion pattern showed in tendency youngest median age (67.0 [Q1–Q3 40.5–76.25], vs. hypoperfusion group 68.0 [Q1–Q3 67.5–70.0], vs. hyperperfusion group 72.0 [Q1–Q3 67.5–79.5], *p* = 0.689). The hypoperfusion group showed a tendency for the longest time-to-recanalization (708 min [Q1–Q3 485.5–751.5], *p* = 0.398), and lowest NIHSS on admission (11.0 [Q1–Q3 9.0–13.5], *p* = 0.697). We observed a tendency for higher age, shortest time-to-recanalization (260.5 min [Q1–Q3 211.0–340.5], *p* = 0.398), highest NIHSS on admission (16.0 [Q1–Q3 11.5–18.0], o = 0.697), smallest difference in NIHSS score after 24 h (3.0 [Q1–Q3 3.0–6.0], *p* = 0.818) and after 3 months from onset (0.5 [Q1–Q3 0.0–3.0], *p* = 0.891) for the hyperperfusion group. In NOVA, considering the quotient of the flow of the affected side divided by the unaffected side at the M1-segment, the highest quotients were reached by the hyperperfusion, and lowest by the hypoperfusion group (1.09 [Q1–Q3 0.94–1.27] vs. 0.77 [Q1–Q3 0.94–1.27], see Table 3).

**Table 1:** Patient demographic and clinical parameters.

	All	Normalized	Hypoperfusion	Hyperperfusion	*p*-value
Number of Patients	18	8	3	7	
Age (median [Q1–Q3])	70.50 [62.25, 76.00]	67.00 [40.50, 76.25]	68.00 [67.50, 70.00]	72.00 [67.50, 79.50]	0.689
Sex [*n* = female (%)]	8 (44.4)	5 (62.5)	1 (33.3)	2 (28.6)	0.383
Intravenous lysis treatment [n (%)]	10 (55.6)	3 (37.5)	3 (100)	4 (57.1)	0.177
NIHSS on admission (median [Q1–Q3])	14.50 [9.52, 16.75]	14.50 [8.00, 17.00]	11.00 [9.00, 13.50]	16.00 [11.50, 18.00]	0.697
Difference NIHSS after 24 h from onset (median [Q1–Q3])	4.00 [3.00, 8.75]	4.50 [2.75, 9.50]	6.00 [4.50, 8.00]	3.00 [3.00, 6.00]	0.818
Difference NIHSS after 3 months from onset (median [Q1–Q3])	1.00 [0.00, 1.75]	1.00 [0.00, 2.50]	1.00 [0.50, 1.00]	0.50 [0.00, 3.00]	0.891
mRS after 36 h from onset (median [Q1–Q3], n)	4.00 [4.00, 5.00] *n* = 17	4.00 [4.00, 4.00] *n* = 8	3.50 (3.25, 3.75] *n* = 3	4.50 [4.00, 5.00] *n* = 6	0.509
mRS after 3 months (median [Q1–Q3], n)	1.00 [1.00, 3.00] *n* = 17	1.00 [0.75, 3.00] *n* = 8	1.00 [1.00, 1.00] *n* = 3	2.50 [2.00, 3.00] *n* = 6	0.169
Intracerebral hemorrhage [n (%)]	11 (61.1)	5 (62.5)	3 (100.0)	3 (42.9)	0.235
Hemorrhagic transformation [n (%)]	12 (66.7)	4 (50.0)	3 (100.0)	5 (71.4)	0.276

The perfusion group with normalized pattern showed youngest age and smallest caliber of occluded artery. The hypoperfusion group showed the lowest NIHSS on admission and highest rate of intracerebral hemorrhage. The hyperperfusion group reached highest age, biggest caliber of the occluded artery and worst clinical outcome with highest NIHSS and mRS. mRS, modified Ranking Score; NIHSS, National Institutes of Health Stroke Scale; Q1–Q3, Quartile one and three.

**Table 2:** Data on treatment modalities and results from angiography.

	All (18)	Normalized (8)	Hypoperfusion (3)	Hyperperfusion (7)	*p*-value
Onset to Recanalization [min] (median [Q1–Q3])	278.00 [215.50, 628.00]	276.00 [216.00, 473.75]	708.00 [485.50, 751.50]	260.00 [211.00, 340.50]	0.398
Duration of mechanical thrombectomy procedure (MT) [min] (median [Q1–Q3])	59.00 [47.00, 85.00]	61.00 [55.25, 70.00]	98.00 [82.50, 109.00]	37.00 [25.25, 51.75]	0.049
Largest occluded artery					0.628
ICA [n (%)]	8 (44.4)	2 (25.0)	2 (66.7)	4 (57.1)	
M1 [n (%)]	7 (38.9)	4 (50.0)	1 (33.3)	2 (28.6)	
M2 [n (%)]	3 (16.7)	2 (25.0)	0 (0.00)	1 (14.3)	
Infarct volume on last available FLAIR follow up (cm^3^ [Q1–Q3], n)	9,429.0 [1,748.0–45,644.0]	4,456.50 [1,493.75–12,660.02]	11,276.0 [10,352.5–28,460.0]	35,278.15 [10,989.08–61,143.50]	0.353
Qualitative level of collaterals					0.657
No n(%)	1 (5.9)	0 (0.0)	0 (0.0)	1 (16.7)	
Minimal n(%)	2 (11.8)	1 (12.5)	0 (0.0)	1 (16.7)	
Moderate n(%)	4 (23.5)	1 (12.5)	1 (33.3)	2 (33.3)	
Good n(%)	10 (58.8)	6 (75.0)	2 (66.7)	2 (33.3)	

The normalized group showed the smallest caliber of occluded artery, while this was the biggest for the hyperperfusion group. The hypoperfusion group showed longest time-to-recanalization, while this was shortest within the hyperperfusion group. ICA, Internal carotid artery; M1, Segment 1 of the medial cerebral artery; M2, Segment 1 of the medial cerebral artery; Q1–Q3, Quartile one and three.

**Table 3:** BOLD-CVR, NOVA parameters and level of collaterals subdivided by perfusion group.

	All	Normalised	Hypoperfusion	Hyperperfusion	*p*-value
BOLD-CVR (ΔBOLD/mmHg) visit one at the affected MCA territory (median [Q1–Q3], n)	0.09 [0.05, 0.12]	0.12 [0.09, 0.19]	0.09 [0.09, 0.11]	−0.01 [−0.02, 0.07]	0.049
Number of Patients visit one at the affected MCA territory n (%)	16 (100)	7 (43.8)	3 (18.8)	6 (37.5)	
BOLD-CVR (ΔBOLD/mmHg) visit two at the affected MCA territory (median [Q1–Q3], n)	0.12 [0.09, 0.17]	0.17 [0.16, 0.18]	0.10 [0.09, 0.11]	0.07 [0.03, 0.10]	0.014
Number of Patients visit two at the affected MCA territory n (%)	12 (100)	5 (41.7)	3 (25)	4 (33.3)	
BOLD-CVR (ΔBOLD/mmHg) visit three at the affected MCA territory (median [Q1–Q3], n)	0.13 [0.12, 0.14]	0.13 [0.12, 0.14]	0.11 [0.11, 0.11]	0.14 [0.07, 0.14]	0.683
Number of Patients visit three at the affected MCA territory n (%)	10 (100)	6 (60)	1 (10)	3 (30)	
NOVA at Visit one Quotient affected and unaffected flow (median [Q1–Q3], n)	0.96 [0.79, 1.21]	1.02 [0.84, 1.19]	0.77 [0.53, 0.90]	1.09 [0.94, 1.27]	0.244
Number of Patients NOVA at Visit one Quotient affected and unaffected flow	17 (100)	8 (47.1)	3 (17.6)	6 (35.3)	
NOVA at Visit two Quotient affected and unaffected flow (median [Q1–Q3], n)	0.97 [0.81, 1.09]	0.82 [0.81, 1.18]	0.63 [0.56, 0.82]	1.09 [1.09, 1.32]	0.136
Number of Patients NOVA at Visit two Quotient affected and unaffected flow	13 (100)	7 (53.8)	3 (23.1)	3 (23.1)	
NOVA at Visit three Quotient affected and unaffected flow (median [Q1–Q3], n)	0.73 [0.71, 0.75]	0.69 [0.69, 0.69]	0.77 [0.77, 0.77]	NA	0.317
Number of Patients NOVA at Visit three Quotient affected and unaffected flow	2 (100)	1 (50)	1 (50)		

BOLD-CVR, NOVA parameters and level of collaterals subdivided by perfusion group. In the visits one and two, negative values within the affected MCA territory in the patients of the hyperperfusion group could be measured. The quotients of the NOVA assessment reflected the qualitative subdivision into the three perfusion patterns, with quotients higher than 1 for the hyperperfusion group and lower than 1, for the hypoperfusion group.

In the follow-up PWI studies, the groups with the normalized perfusion after MT (Figure 2) retained their normalized perfusion pattern over all follow-ups. Similarly, for the hypoperfusion group (Figure 3), we observed a persistent circumscribed hypoperfused area without detectable changes in any follow-up. There was no case with later reperfusion. In contrast, the hyperperfusion group showed a tendency to develop a hypoperfusion in the initially affected hyperperfused area between visit two and three (7 days (+/− 2 days) and 90 days (+/− 14 days), see Figure 3), with respective decreased CBF, CBV and increased Tmax values.

The final infarct volume was estimated by quantifying lesion volume from the fluid-attenuated inversion recovery (FLAIR) at visit 3. A tendency for the largest infarct volumes were found in the hyperperfusion group, followed by the hypoperfusion group and the normalized group (35,278 cm^3^ [Q1–Q3 10,989–61,143], 11,276 cm^3^ [Q1–Q3 10,352–28,460 cm^3^] and 4,456.6 cm^3^ [Q1–Q3 1,493–12,660 cm^3^], respectively, *p* = 0.353, see Table 2). Also, we saw a tendency for a better collateral status for the “normalized” group, while this was the poorest for the hyperperfusion group (good status in normalized group for 6 (75%) patients, vs. 2 (33%) in hyperperfusion group, *p* = 0.657).

In Table 3 we summarized the results of the advanced perfusion studies with BOLD-CVR and NOVA. Within the BOLD-CVR analysis, we observed the highest values in the normalized group for the affected medial cerebral artery (MCA) region, while lower and even negative values were observed at visit one for the hyperperfusion group (0.12 [Q1–Q3 0.09–0.19] vs. − 0.01 [Q1–Q3–0.02-0.07], *p* = 0.049).

## 4 Discussion

There is an urgent need to better understand RF after treatment of LVO stroke, in order to enhance available therapeutic options. In this study, we investigated the phenomenon of RF in patients with LVO after successful MT by applying advanced imaging techniques at different time points before and after MT, including PWI, BOLD-CVR and NOVA. Qualitatively, we distinguished between three different reperfusion patterns in the affected stroke area after successful MT: hypo-, hyper-, and normalized perfusion. This qualitative subdivision was quantitatively reflected by the NOVA-analysis, where highest quotients were reached in the hyperperfusion, and lowest in the hypoperfusion group. Repeated PWI studies showed “static” patterns for the hypo- and normalized perfusion cohort, meaning that perfusion remained either normal or below the contralateral side (hypoperfusion) between visits 1 and 3 (up to day 90). However, in the hyperperfusion cohort, we observed a transformation of the hyperperfused infarct area into a hypoperfusion pattern up to visit three. In line with this, we observed that both, the hypo- and hyperperfusion cohorts, tended to have worse clinical outcomes compared to the group with normalized perfusion. Collateral-status, time-to-recanalization, and door-to-groin appeared to be associated with certain post-MT perfusion patterns, even though this did not reach statistical significance, most likely due to the very small patient groups. In this regard, the hyperperfusion group showed a tendency for the worst collateral status and shortest time-to-recanalization, while the hypoperfusion group had the second worst collateral status and the longest time-to-recanalization. Strikingly, the duration of the MT-procedure was significantly shorter in the hyperperfusion group, compared to the other groups.

### 4.1 Normalized perfusion cohort

Normalized perfusion after successful MT correlates with a favorable clinical outcome (14). This cohort presented accordingly a trend for the lowest mRS score in the 3 months follow up. Collateral status tended to be better and final infarct volume to be smaller. As potential confounders, younger age, more distal artery occlusions, and lower NIHSS at onset were found on average in this group of patients. This favorable clinical and radiological outcome is consistent with the results of previous studies (16, 17).

### 4.2 Hypoperfusion cohort

Patients presenting with hypoperfusion post-MT showed a tendency toward the second-worst clinical outcome. They also had the longest time-to-recanalization. Accordingly, in previous studies, RF was mostly linked to persistent hypoperfusion associated with longer onset-to-arrival and time-to-recanalization (11, 18–20). Additionally, the duration of the MT was significantly longer in patients with hypoperfusion, potentially indicating difficult interventons with increased likelihood of residual vessel obstructions or fragment embolism. In accordance with Ng et al., we also observed the highest rate for hemorrhagic transformation in this group (11). In our cohort, this was the group with most patients receiving IVT treatment.

This group also displayed a trend for poor leptomeningeal collaterals compared to the normalized group, which was previously suggested to be associated with RF (21) and generally perceived as an independent predictor for poor clinical outcome (3, 22).

### 4.3 Hyperperfusion cohort

As one key finding of our study, we found a tendency for the least clinical improvement and poorest clinical outcome within the hyperperfusion group. This cohort also presented a trend for the worst collateral status, largest infarct volumes, and, interestingly, shortest MT-duration and time-to-recanalization. In general, these three perfusion patterns have been previously described solely after thrombolysis, therefore not exclusively in patients with LVO after MT and only within a frame of 24 h after treatment (23). The poor clinical outcome and clinical recovery could be attributed to higher age, more proximally affected vessels, along with higher baseline NIHSS, and larger infarct volumes. We also observed the smallest difference in NIHSS 24 h after onset in this group, even though none of these values reached statistical significance. These findings are in accordance with Shimonaga et al.’s (19). Potreck et al. came to an opposite conclusion: their hyperperfusion cohort showed a similar outcome compared with their normalized perfusion cohort. This difference could be explained by the different assessment time points of our studies. While their study cohorts were evaluated based on perfusion patterns obtained within 24 h after successful recanalization, our analysis is based on perfusion data acquired within 3 days post-recanalization and includes follow-up until day 90. Their hyperperfusion cohort included patients with temporary hyperperfusion, with a potential to normalization within 18–36 h after MT (17). However, we observed that hyperperfusion post-MT resulted in a long-term hypoperfusion, which was associated with poor clinical outcome, and indicating that any post-MT perfusion alterations may be associated with a worse clinical outcome (11, 24, 25). Therefore, this study suggests that hypo- and hyperperfusion should both be perceived as RF, indicating a poor clinical outcome. In this regard, Lin et al. have shown the importance of post-MT PWI imaging to recognize hyperperfusion and its consequences (26). Our results emphasize the importance of advanced imaging up to 3 months post-MT since hypoperfusion, as a sign of RF, might appear also after such a latency. Accordingly, identifying patients with greater risk for poor outcome could be enabled by advanced imaging.

In our study, hyperperfusion was not significantly correlated with shorter onset-to-recanalization, indicating that merely reducing time-to-recanalization does not prevent RF. Similarly, Shimonaga et al. could not find a significant difference, even though they described a tendency toward a longer time to recanalization (19).

### 4.4 Pathophysiology of RF and possible therapeutic implementations

As shown in rodent models, leptomeningeal collaterals are crucial for the survival of at-risk-tissue (27). Preclinical data suggest that fast, overshooting reperfusion after recanalization in stroke is linked to poor leptomeningeal collaterals and reperfusion injury (28), which was also reflected in our patients with altered perfusion patterns. To this regard, the reperfusion injury describes a damage occurring at the vascular level due to reperfusion. The exact pathophysiological mechanisms are not fully understood yet. One of the most accepted theories proposes that following a vessel occlusion, endothelial damage at the level of the smooth muscle cells may lead to an impaired autoregulation and thus to an increased rigidity of the cerebral vessels. As a consequence, alteration of the cerebral perfusion pressure may favor increased blood–brain barrier permeability, thus favoring hemorrhagic transformation (26, 29). Also, increased vessel rigidity and a damaged endothelium, to which a thrombus might attach with greater difficulty, could explain the shorter duration of MT in the hyperperfusion group. Another theory suggests a damaging effect of free radicals during hypoxic phases, leading to altered endothelium function, vasodilatation, and in a later phase to pericyte contraction, and increased blood–brain-barrier permeability (8, 26), which also possibly could reflect the changes in the perfusion studies in our hyperperfusion group between visit one and three. A relevant role could play the reactive hyperemia, in which a phase of cerebral ischemia is followed by a transient increase in perfusion due to autoregulatory mechanisms. This phenomenon has been associated with both favorable prognostic implications (30) and adverse outcome (31) However, this does not explain the long-term differences observed at Visit two and three. Similarly, the so-called luxury perfusion, which frequently appears after restored blood-supply, may explain a perfusion alteration in the subacute phase of a stroke or after MT respectively, but not the persistent changes in the affected area (32–34).

In addition, due to mechanical stress, MT itself may lead to additional damage of the endothelium of the vessel wall (35). Damaged endothelium activates thrombocytes and potentially triggers an immune response involving neutrophils, leucocytes, and monocytes (7, 10, 35, 36), and therefore leading to a malfunction of autoregulation.

In accordance with some other studies, our dataset shows an increased rate of hemorrhagic transformation in the hyperperfusion cohort (19, 29, 37–41). Additionally, our study underlines poor clinical outcome in this group. Hypoperfusion in the affected area at visit three followed the initial hyperperfusion. To the best of our knowledge, this tendency has not been described before and emphasizes the risk of hyperperfusion post-MT.

As mentioned above, patients exhibiting hypoperfusion may have experienced residual distal vessel occlusion due to distal embolization. In such cases, a timely repeated attempt with MT and/or local application of thrombolysis could be beneficial. Given the not specified pathophysiology of the phenomenon, it is not possible to implement a clear therapeutic strategy in the case of the hyperperfusion group.

Also, some therapeutic agents have been tested in rodent models, to counteract some proposed mechanism responsible for the reperfusion failure. Therefore, Yang et al. tested an agent called NA-1 which successfully inhibited ischemia-induced pericyte constriction (42). Regarding the neutrophilic stalls, El Amki et al. successfully applied anti-Ly6G-antibodies in rodent models, which hindered the adhesion of neutrophils, preventing perfusion disturbances in recanalized vessels (7), thus providing a proof-of-concept for new therapeutic strategies.

### 4.5 Advanced perfusion studies

BOLD-CVR displayed significant differences between the reperfusion groups, thus proving a suitable and sensitive tool for impaired cerebral reperfusion. Both, hypo- and hyperperfusion post-MT correlated with a reduced cerebrovascular reserve in BOLD-CVR imaging. Using the same data set, another analysis suggested poor clinical outcome 3 months post intervention in patients with impaired BOLD-CVR (43). Therefore, BOLD-CVR might be useful as an early-stage predictor of reperfusion failure and poor clinical outcomes post-MT (27, 44).

### 4.6 Strengths and limitations

In this study, we used advanced hemodynamic imaging techniques to identify three different post-MT perfusion patterns in patients with LVO over a period of 3 months. Previous studies focused on either hyperperfusion (19, 26, 29) or hypoperfusion (11, 45) without comparing them, and only applied advanced imaging shortly after stroke (11, 19, 26, 27, 29, 45). Thus, we were able to unveil the course of tissue reperfusion after MT, particularly finding that initial hyperperfusion was followed by hypoperfusion and accompanied by reduced CVR.

However, our study has several limitations. One important limitation is the small number of patients, impeding proper statistical analysis. The small number resulted from a relatively high rate of withdrawal during the acute enrolment phase, as well as from frequently incomplete datasets, either due to missing perfusion studies prior to MT or the complexity of certain acquisitions, such as BOLD-CVR, which requires active patient participation. Furthermore, we only analyzed patients fulfilling the criteria for a successful MT (TICI 2b-3), in order to study RF after successful recanalization, which however, next to the non-randomized nature of the cohort, limits generalization of findings to other patients. While we performed a qualitive analysis of post-MT perfusion using perfusion imaging, further studies could include additional quantitative perfusion analysis of the interested areas.

## 5 Conclusion

In patients with LVO stroke, both, hypo- and hyperperfusion after successful MT potentially indicate poor clinical outcome. Thus, not only hypo-, but also hyperperfusion should be considered as a manifestation of RF. Good collaterals are associated with healthy reperfusion and favorable outcome. BOLD-CVR could be used after MT to anticipate a RF and to better estimate prognosis in patients after MT. Further studies may further illuminate the pathophysiological mechanisms behind this phenomenon.

## Data Availability

The raw data supporting the conclusions of this article will be made available by the authors, upon reasonable request.

## Glossary

ADC: Apparent Diffusion Coefficient
BOLD-CVR: Blood Oxygenation-Level dependent Cerebrovascular Reactivity
CBF: Cerebral Blood Flow
CBV: Cerebral Blood Volume
DWI: Diffusion Weighted Imaging
FLAIR: Fluid-Attenuated Inversion Recovery
IMPreST: Interplay of Microcirculation and Plasticity after ischemic STroke
IVT: IntraVenous Thrombolysis
LVO: Large Vessel Occlusion
MCA: Medial Cerebral Artery
mRS: modified Ranking Score
MT: Mechanical Thrombectomy
MTT: Mean Transit Time
MPRAGE: T1 Magnetization-Prepared Rapid Gradient Echo
NIHSS: National Institutes of Health Stroke Scale
PWI: Perfusion Weighted Imaging
Q1–Q3: Quartiles 1 and 3
RF: Reperfusion Failure
TR/TE: Repetition and Echo Time
TICI: Thrombolysis in Cerebral Infarction
Tmax: Time-to-Maximum
TTP: Time-To-Peak
